# Two Cases of Subarachnoid Hemorrhage Associated With COVID-19 Infection Yielding Favorable Functional Outcomes

**DOI:** 10.7759/cureus.97791

**Published:** 2025-11-25

**Authors:** Sayaka Terazono, Yuki Sakaeyama, Ryo Matsuzaki, Taiki Tokuyama, Nobuo Sugo

**Affiliations:** 1 Department of Neurosurgery, Faculty of Medicine, Toho University, Tokyo, JPN

**Keywords:** covid-19, favorable outcome, severe acute respiratory syndrome coronavirus 2, subarachnoid hemorrhage, vertebral artery dissecting aneurysm

## Abstract

Subarachnoid hemorrhage (SAH) is a life-threatening cerebrovascular disorder characterized by high mortality and unfavorable long-term outcomes. During the COVID-19 pandemic, SARS-CoV-2 infection has been associated with systemic inflammation and coagulopathy, which may result in cerebrovascular complications such as intracranial hemorrhage (ICH). However, the characteristics and outcomes of COVID-19-associated SAH remain unclear, and favorable outcomes are rarely reported. We encountered two male patients with COVID-19-associated SAH, one with a non-aneurysmal SAH and another with a ruptured vertebral artery dissecting aneurysm, both of whom achieved favorable recovery following appropriate management. These cases suggest that early diagnosis, prevention of rebleeding, and timely standard treatment can lead to good outcomes even in COVID-19-associated SAH.

## Introduction

Subarachnoid hemorrhage (SAH) accounts for 1-6% of all strokes and is characterized by high mortality during the acute phase and unfavorable long-term functional outcomes [1]. Although recent studies have described intracranial hemorrhage (ICH) and other cerebrovascular events in association with COVID-19, a causal relationship between SARS-CoV-2 infection and SAH remains unproven and controversial [1]. This ongoing uncertainty provides the rationale for reporting individual cases to better characterize possible clinical patterns and outcomes.

During the pandemic, COVID-19 caused by SARS-CoV-2 has been shown to induce systemic inflammation and coagulopathy, contributing to the development of cerebrovascular disorders; however, the cases presented in this report did not exhibit such abnormalities, suggesting that other mechanisms or a coincidental relationship may be involved. Recent studies have also reported ICH following messenger ribonucleic acid (mRNA) COVID-19 vaccination, which may share similar inflammatory and vascular mechanisms with COVID-19 infection itself [2]. ICH is a clinically significant cerebrovascular complication in COVID-19 patients, with high mortality and unfavorable outcomes [3-5]. This association has been attributed to SARS-CoV-2-induced systemic inflammation, endothelial dysfunction, and coagulopathy, which may increase vascular fragility and bleeding risk [5].

At our institution, COVID-19 testing (PCR or antigen) has been routinely conducted for all hospitalized patients, including those with SAH, since the beginning of the pandemic. SAH occurring during the active phase of confirmed COVID-19 infection has been regarded as COVID-19-associated SAH, based on the temporal rather than causal relationship between the two conditions. However, the clinical characteristics and outcomes of COVID-19-associated SAH remain unclear, and reports of favorable outcomes are rare.

Although most reports of COVID-19-associated SAH have described severe cases with poor outcomes, evidence regarding mild cases with favorable recovery is scarce.

The rationale of this report is to emphasize that, although most COVID-19-associated SAH cases reported to date have had severe disease and poor outcomes, mild cases with favorable recovery can also occur. Clarifying such clinical variations may help refine our understanding of the pathophysiological spectrum and management strategies of COVID-19-associated SAH. In this report, we describe two cases of SAH that occurred during the clinical course of confirmed COVID-19 infection, suggesting a possible temporal association between the two conditions. Both patients had mild disease severity and achieved favorable functional outcomes following appropriate management.

## Case presentation

Case 1

A 72-year-old retired man presented with left upper limb numbness as the chief complaint. He had a medical history of angina pectoris (after percutaneous coronary intervention), hypertension, and dyslipidemia, and was on aspirin treatment. He was a non-smoker and reported no alcohol or illicit drug use. There was no family history of cerebrovascular disease and no evidence of traumatic brain injury.

At admission, he presented with left upper limb numbness. His neurological findings were Glasgow Coma Scale (GCS) E4V5M6 and World Federation of Neurosurgical Societies grade 1 (WFNS grade 1). He had developed a fever the day before admission; therefore, a COVID-19 antigen test was performed upon admission as part of our institutional screening policy for all hospitalized patients, which confirmed SARS-CoV-2 infection. Chest CT showed no evidence of pneumonia. Blood tests revealed D-dimer 0.5 μg/mL, CRP 0.1 mg/dL, and platelets 18.1 × 10⁴/μL, indicating no inflammation or coagulopathy. He was treated with remdesivir for COVID-19.

**Figure 1:**
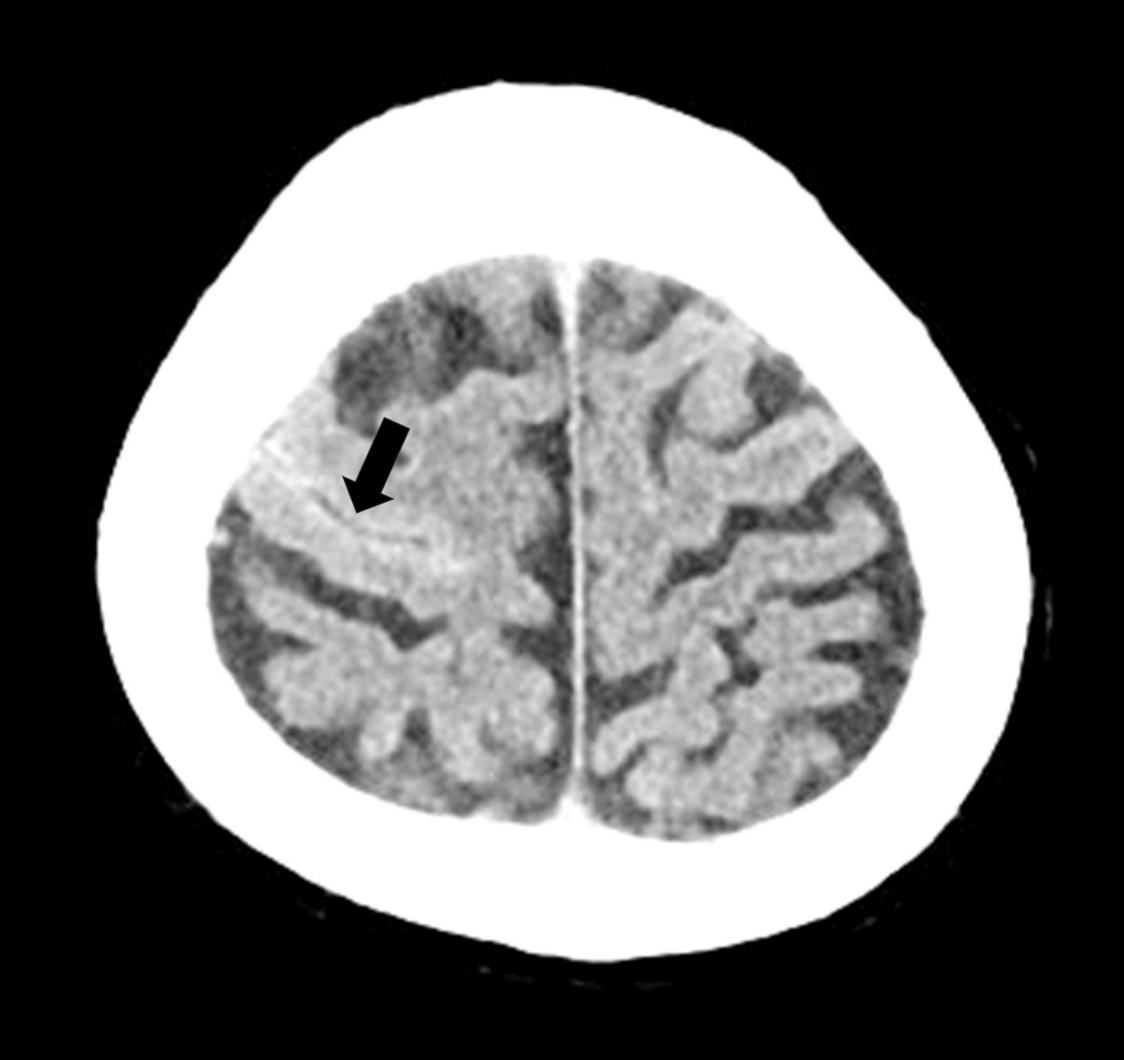
Head CT (Case 1). Non-contrast-enhanced axial head CT on admission showing localized subarachnoid hemorrhage centered around the right precentral sulcus (black arrow).

Non-contrast-enhanced axial head CT on admission revealed localized SAH centered around the right precentral sulcus (Figure 1). No ischemic lesions were observed.

His left upper limb numbness was considered to result from transient cortical irritation involving the right precentral and postcentral regions caused by localized subarachnoid blood, without evidence of cortical or ischemic lesions on CT or MRI. This presentation was regarded as atypical for non-aneurysmal SAH.

**Figure 2:**
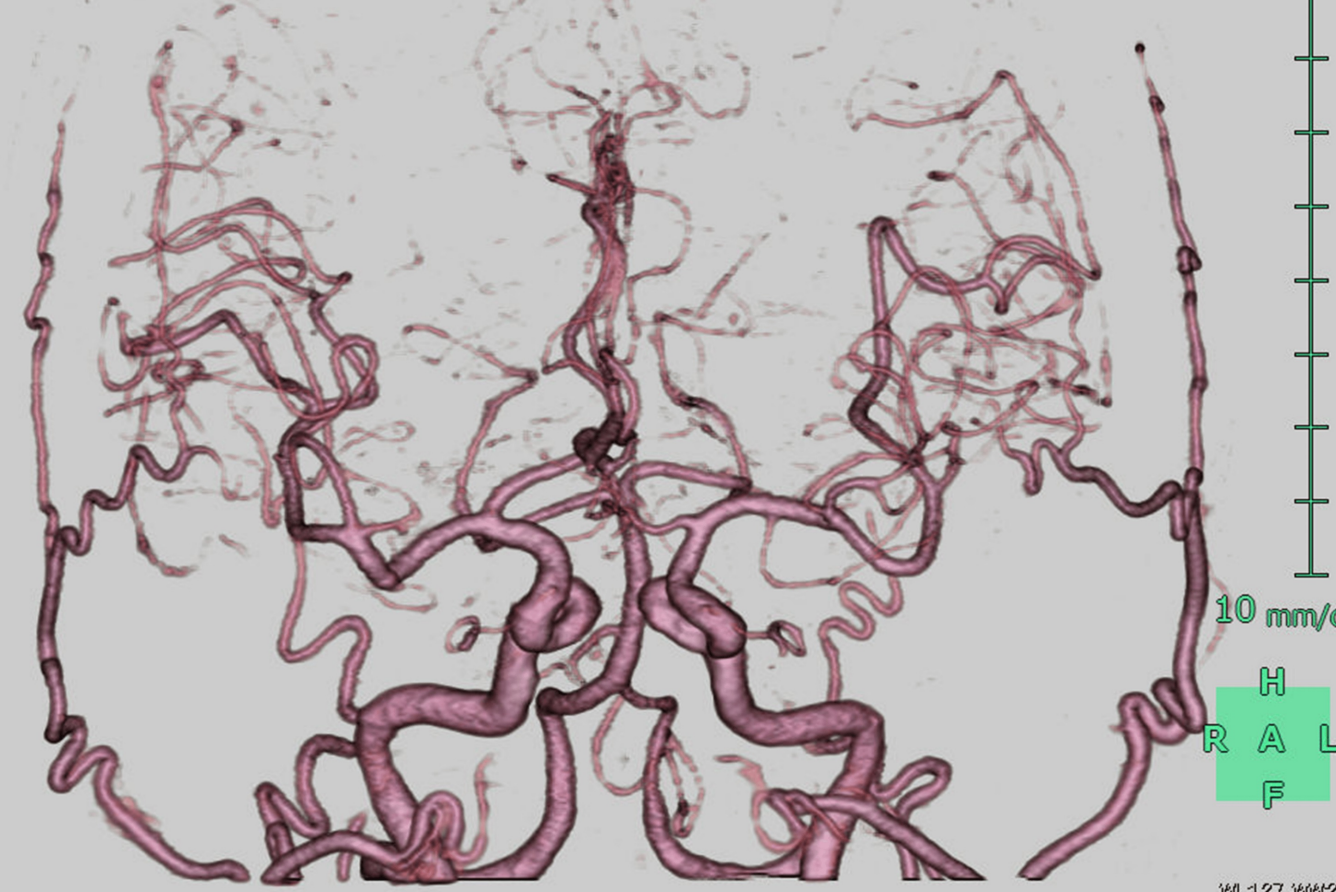
Three-dimensional computed tomography angiography (3D-CTA) (Case 1). No cerebral aneurysms or other vascular abnormalities were observed. Note: The blue color indicates orientation markers (anterior–posterior and right–left directions) and does not represent any pathological findings.

**Figure 3:**
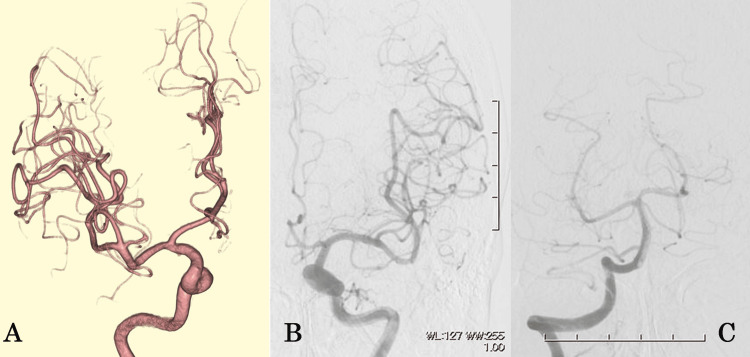
Digital subtraction angiography (DSA) (Case 1). (A) Right internal carotid artery; (B) left internal carotid artery; (C) right vertebral artery.

No cerebral aneurysms or vascular abnormalities were observed on three-dimensional computed tomography angiography (3D-CTA) (Figure 2) or digital subtraction angiography (DSA) (Figure 3A-3C).

Follow-up 3D-CTA was performed on days 3 and 6 to exclude delayed aneurysm formation or vascular abnormality, which can occasionally occur in angiogram-negative SAH. Each study was conducted with a reduced contrast dose and adequate hydration, and no evidence of vasospasm or new lesions was observed.

He was diagnosed with non-aneurysmal SAH. He remained neurologically stable and was discharged on day 10. Follow-up at one month revealed no recurrence or complications. The modified Rankin Scale (mRS) score at discharge was 0.

Case 2

**Figure 4:**
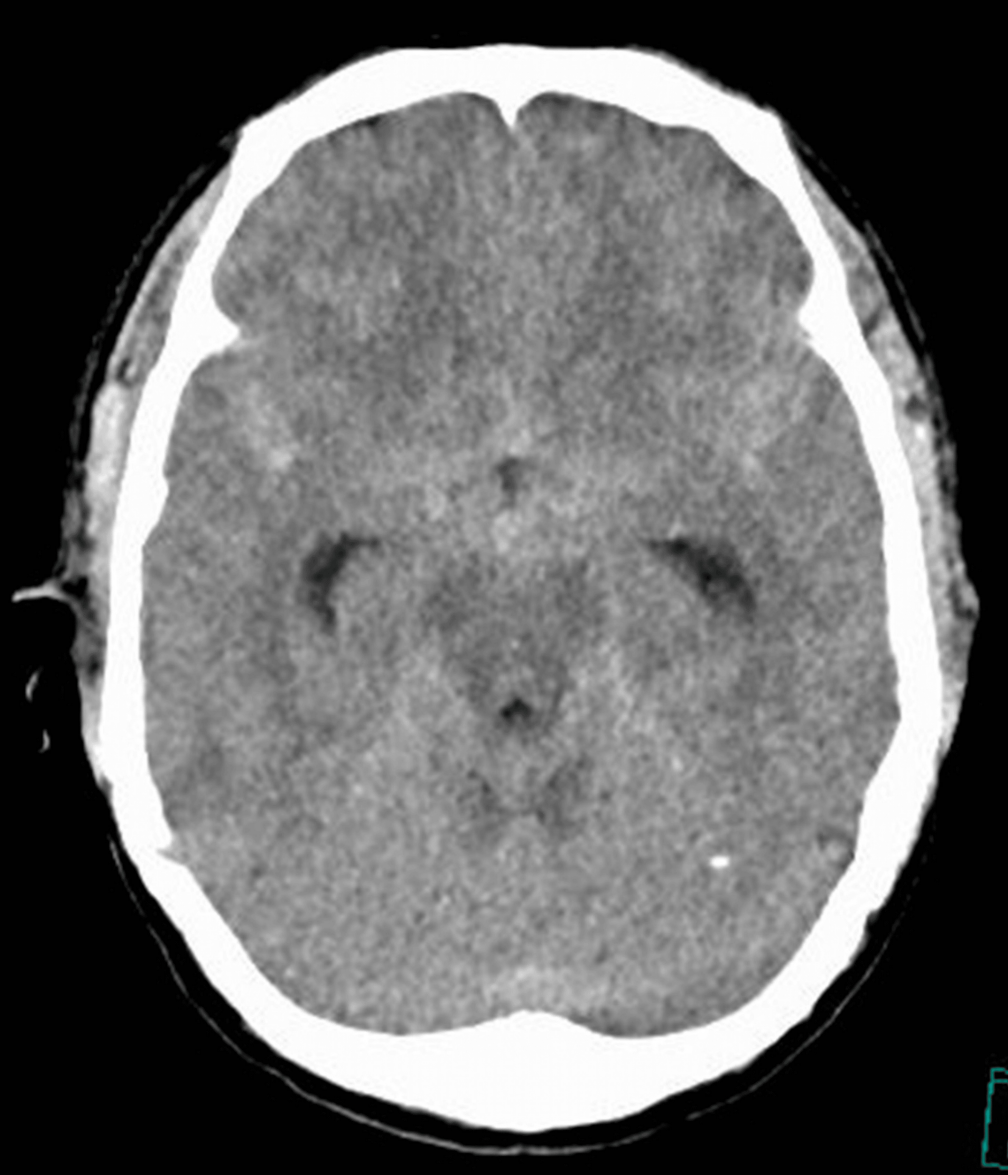
Head CT (Case 2). Non-contrast-enhanced axial head CT showing diffuse subarachnoid hemorrhage involving the basal and ambient cisterns.

A 35-year-old man on maintenance hemodialysis for childhood-onset nephrotic syndrome presented to the ED with sudden-onset severe headache, which was his chief complaint. He worked as a medical engineer and lived independently. He was a non-smoker and rarely consumed alcohol, with no history of recreational drug use. There was no family history of cerebrovascular or renal disease. Non-contrast-enhanced axial head CT obtained on the day of onset revealed diffuse SAH predominantly involving the basal and ambient cisterns (Figure 4), with mild dilation of the temporal horns suggesting early hydrocephalus.

**Figure 5:**
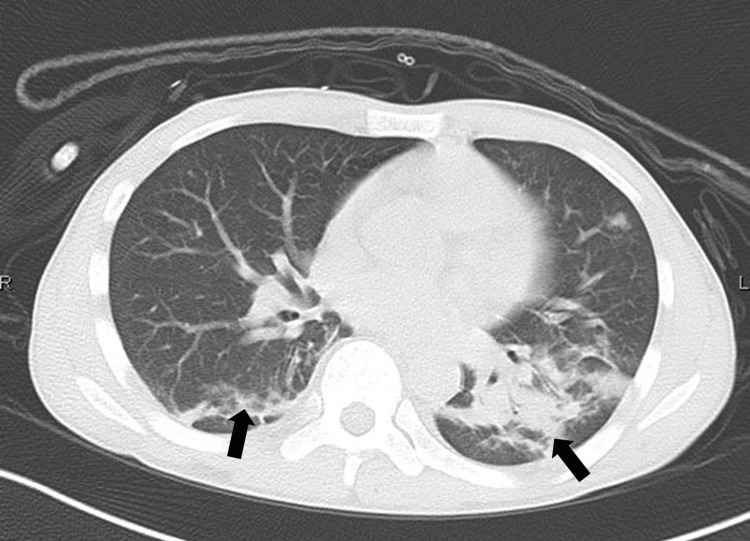
Chest CT (Case 2). Chest CT showing pneumonia with ground-glass opacities.

No intraparenchymal or ischemic lesions were observed. On arrival, his neurological status was assessed as GCS E4V5M6 and WFNS grade 1. He had developed a fever four days before presentation, and a positive qualitative SARS-CoV-2 antigen test confirmed COVID-19. Chest CT revealed bilateral ground-glass pneumonia (Figure 5).

He was treated with remdesivir for COVID-19. Blood tests revealed D-dimer 0.5 μg/mL, CRP 1.3 mg/dL, and fibrinogen 331 mg/dL, indicating a mild inflammatory response without coagulopathy.

Contrast injection was performed via the left vertebral artery during DSA for detailed vascular evaluation.

**Figure 6:**
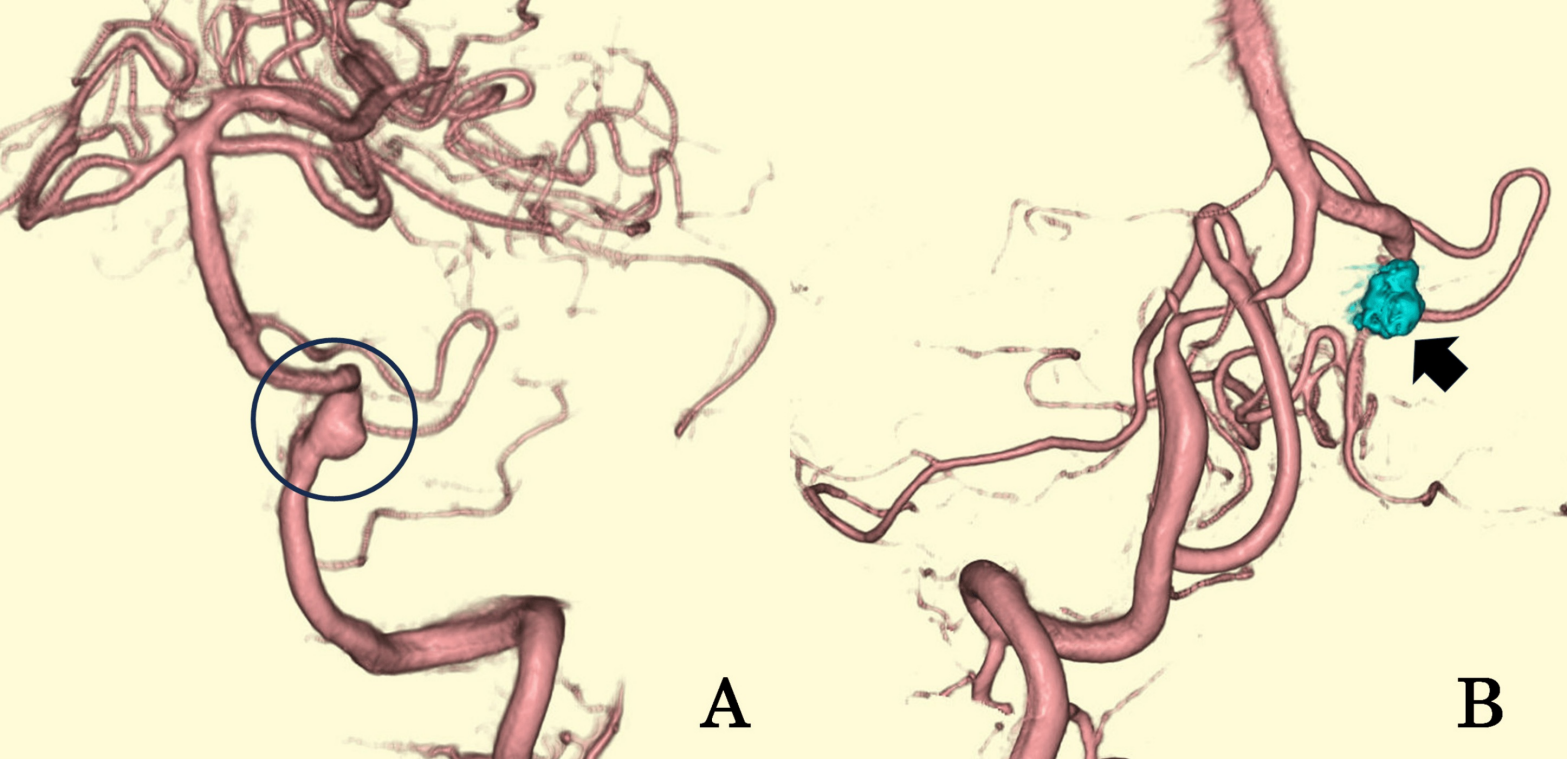
Digital subtraction angiography (DSA) and 3D reconstructed images (Case 2). (A) Digital subtraction angiography with 3D reconstruction showing a 5.5-mm left vertebral artery dissecting aneurysm (circle). (B) Postoperative angiogram demonstrating complete occlusion of the parent artery (arrow). Note: The blue color indicates orientation markers (anterior–posterior and right-left directions) and does not represent any pathological findings.

DSA showed a 5.5-mm left vertebral artery dissecting aneurysm (VADA) located distal to the origin of the posterior inferior cerebellar artery (PICA) (Figure 6A). The contralateral (right) vertebral artery was dominant, while the left vertebral artery showed normal caliber without evidence of dissection or stenosis. DSA with contrast injection was performed immediately before a scheduled hemodialysis session to ensure effective clearance of the contrast agent. No dialysis-related complications or adverse reactions occurred. Endovascular surgery was performed the same day to achieve complete occlusion of the parent vertebral artery (Figure 6B).

For anticoagulation management during hemodialysis, heparin was discontinued, and antiplatelet agents were administered perioperatively. Postoperative follow-up DSA and 3D-CTA showed no parent artery recanalization or cerebral vasospasm. The patient remained alert, and the ventricular enlargement improved spontaneously without cerebrospinal fluid drainage or shunt intervention. He was discharged after one month with mild short-term memory impairment as a sequela and an mRS score of 1.

## Discussion

COVID-19-associated SAH is rare, with an incidence of approximately 0.1%, but has a reported mortality rate of 40.8% [3]. In particular, non-aneurysmal SAH cases have an extremely high mortality rate of 85.7%, with worse outcomes than aneurysmal SAH [3]. In the early phase of the pandemic, non-aneurysmal SAH cases were relatively common [6], and even in reports from 2021-2022, they accounted for more than half of SAH cases (8 out of 14) [7]. This may be associated with anticoagulant therapy and extracorporeal membrane oxygenation (ECMO) use in severe COVID-19 cases, which increases the risk of hemorrhagic events. The outcomes of COVID-19-associated SAH depend on the patient’s general condition, COVID-19 severity, SAH subtype (aneurysmal SAH, non-aneurysmal SAH, or dissecting lesions), and intervention timeliness. High-mortality cases are presumed to predominantly involve severe COVID-19 requiring anticoagulant therapy or ECMO [3-5]. In addition, pandemic-related changes in patients’ healthcare-seeking behavior, referral and admission criteria, and the strain on medical resources may have caused selection bias in case reporting [3].

Whether COVID-19 directly increases the aneurysmal SAH incidence or rupture risk remains controversial. Although inflammation, coagulopathy, and endothelial dysfunction have been suggested to be involved, a causal relationship has not been proven [8]. Batcik OE et al. reported that COVID-19 can cause SAH and emphasized the need to clarify its pathophysiological mechanisms early [9]. Chong PF et al. indicated that COVID-19 might activate macrophages and T lymphocytes, continuously increasing inflammatory cytokines such as IL-6 and tumor necrosis factor-α, which could weaken cerebral vessels and cerebral aneurysm walls and increase the rupture risk [10]. A large-scale analysis of over 280,000 cases by Qureshi AI et al. demonstrated a trend toward increased SAH risk in patients with COVID-19 [11]. Furthermore, Dodd WS et al. analyzed 10 aneurysmal SAH cases during the COVID-19 pandemic and reported “atypical” features: small size (≤4 mm), a high proportion of dissecting or pseudoaneurysms (40%), and a predominance in the posterior circulation [6]. Specifically, ruptured VADA and other dissecting lesions have attracted clinical attention [12,13].

Both cases were mild (WFNS grade 1): one was treated conservatively, and the other underwent early endovascular coil embolization. Both patients were discharged walking independently. Although COVID-19-associated SAH has generally been linked to a high mortality rate [3], these two mild cases indicate that favorable outcomes may occur with timely diagnosis and appropriate management.

In our cases, a causal link between SARS-CoV-2 infection and SAH cannot be inferred. Laboratory data in both patients showed no significant systemic inflammation or coagulopathy (e.g., normal or only mildly elevated D-dimer and CRP), further weakening a mechanistic argument. Second, confounding factors exist: in Case 1, ongoing aspirin therapy may have increased bleeding susceptibility; in Case 2, a vertebral artery dissecting aneurysm, a lesion known to rupture spontaneously, provides a sufficient alternative explanation. Third, the available data do not fulfill Bradford Hill criteria (e.g., strength, consistency, temporality beyond coincidence, biological gradient) [14]. Accordingly, our use of the term “COVID-19-associated SAH” is operational and temporal, denoting occurrence during active infection rather than implying causality. While prior literature proposes plausible pathways (inflammation, endothelial dysfunction, hypercoagulability), such mechanisms were not demonstrable in these patients. Future studies with appropriate controls, serial inflammatory and coagulation biomarkers (e.g., IL-6, CRP, D-dimer), and vessel-wall imaging could better delineate whether a subset of SAH during COVID-19 reflects causal biology versus coincidence. These findings should be interpreted as descriptive clinical observations that may help characterize the milder end of the disease spectrum rather than establish new prognostic conclusions.

Future prospective studies that consider disease severity and treatment strategies are needed to further clarify the association between COVID-19 and SAH. Recent case reports, including those by Yangi K et al. (2023) and others, have described intracranial hemorrhages, including SAH, following COVID-19 infection or vaccination, suggesting multifactorial vascular involvement. A summary of previously reported COVID-19-associated SAH cases is presented in Table 1.

**Table 1:** Summary of previously reported cases of subarachnoid hemorrhage (SAH) associated with COVID-19 infection or vaccination. The table summarizes patient characteristics, SAH subtype, treatment, and outcomes reported in the literature, including the two cases presented in this study.

Author (Year)	Type of Hemorrhage	COVID-19/Vaccine Association	Treatment	Outcome (mRS)
Dodd WS et al., 2021 (World Neurosurg) [6]	Aneurysmal SAH	COVID-19 infection	Endovascular coiling	4
Khan D et al., 2022 (Pathogens) [7]	Ruptured aneurysm	COVID-19 infection	Clipping	3
Sato T et al., 2022 (Brain Hemorrhages) [12]	Vertebral artery dissecting aneurysm	Severe COVID-19	Parent artery occlusion	2
Chong PF et al., 2024 (BMC Neurology) [10]	Aneurysm rupture with inflammation	COVID-19 infection	Conservative	4
Yangi K et al., 2023 (Cureus) [2]	Intracranial hemorrhage post-mRNA vaccination	Post-vaccine	Conservative	2
Present cases (2025)	SAH (non-aSAH, VADA)	COVID-19 infection	Conservative / Endovascular	0 / 1

## Conclusions

We report two mild cases of COVID-19-associated SAH with favorable outcomes following prompt, standard management. Although COVID-19-associated SAH is generally linked to high mortality, these findings should be interpreted as limited clinical observations suggesting that mild cases may achieve good recovery with appropriate care.

## References

[1] Cornea A, Simu M, Rosca EC (2022). Subarachnoid hemorrhage in patients with SARS-CoV-2 infection: protocol for a scoping review. Brain Sci.

[2] Yangi K, Demir DD, Uzunkol A (2023). Intracranial hemorrhage after Pfizer-BioNTech (BNT162b2) mRNA COVID-19 vaccination: a case report. Cureus.

[3] Lim MJ, Yeo J, Fong KY (2023). Characteristics of subarachnoid hemorrhage associated with COVID-19 infection: a systematic review and descriptive analysis. J Stroke Cerebrovasc Dis.

[4] Schmidbauer ML, Ferse C, Salih F (2022). COVID-19 and intracranial hemorrhage: a multicenter case series, systematic review and pooled analysis. J Clin Med.

[5] Daly SR, Nguyen AV, Zhang Y, Feng D, Huang JH (2021). The relationship between COVID-19 infection and intracranial hemorrhage: a systematic review. Brain Hemorrhages.

[6] Dodd WS, Jabbour PM, Sweid A (2021). Aneurysmal subarachnoid hemorrhage in patients with coronavirus disease 2019 (COVID- 19): a case series. World Neurosurg.

[7] Khan D, Naderi S, Ahmadi M, Ghorbani A, Cornelius JF, Hänggi D, Muhammad S (2022). Intracranial aneurysm rupture after SARS-CoV-2 infection: case report and review of literature. Pathogens.

[8] Fiani B, Fowler JB, Figueras RA, Hessamian K, Mercado N, Vukcevich O, Singh MK (2021). Ruptured cerebral aneurysms in COVID-19 patients: a review of literature with case examples. Surg Neurol Int.

[9] Batcik OE, Kanat A, Cankay TU (2021). COVID-19 infection produces subarachnoid hemorrhage; acting now to understand its cause: a short communication. Clin Neurol Neurosurg.

[10] Chong PF, Higashi K, Matsuoka W (2024). Persistent intracranial hyper-inflammation in ruptured cerebral aneurysm after COVID-19: case report and review of the literature. BMC Neurol.

[11] Qureshi AI, Baskett WI, Huang W (2021). Subarachnoid hemorrhage and COVID-19: an analysis of 282,718 patients. World Neurosurg.

[12] Sato T, Miura Y, Yasuda R, Toma N, Suzuki H (2022). Vertebral artery dissecting aneurysm rupture under severe COVID-19. Brain Hemorrhages.

[13] Nakamura Y, Takashima C, Nonaka T (2023). Early recanalization and vasospasm after endovascular treatment in a case of ruptured vertebral artery dissecting aneurysm associated with COVID-19. Surg Neurol Int.

[14] Hill AB (1965). The environment and disease: association or causation?. Proc R Soc Med.

